# Chronic Nitrite Nitrogen Responses in *Penaeus monodon*: From Growth Phenotypes to Metabolic Hub Signaling

**DOI:** 10.3390/biology15201795

**Published:** 2026-10-09

**Authors:** Jianzhi Shi, Li Zhang, Song Jiang, Jianhua Huang, Lishi Yang, Yundong Li, Yafei Duan, Yangyang Ding, Xu Chen, Qibin Yang

**Affiliations:** 1 Key Laboratory of Efficient Utilization and Processing of Marine Fishery Resources of Hainan Province, Sanya Tropical Fisheries Research Institute, Sanya 572018, China; 2 Key Laboratory of South China Sea Fishery Resources Exploitation and Utilization, Ministry of Agriculture and Rural Affairs, South China Sea Fisheries Research Institute, Chinese Academy of Fishery Sciences, Guangzhou 510300, China; 3 State Key Laboratory of Biocontrol, Southern Marine Science and Engineering Guangdong Laboratory (Zhuhai), School of Life Sciences, Sun Yat-sen University, Guangzhou 510275, China; 4 Department of Ecology and Institute of Hydrobiology, Jinan University, Guangzhou 510632, China

**Keywords:** *Penaeus monodon*, nitrite nitrogen, chronic toxicity, growth inhibition, transcriptomics, tissue-specific

## Abstract

Nitrite nitrogen is a prevalent environmental stressor in intensive shrimp aquaculture that impairs growth and health, yet its chronic effects and underlying molecular mechanisms in *Penaeus monodon* remain incompletely understood. To address this, we exposed juvenile *P. monodon* (initial body weight 2.06–2.34 g) to 0, 4, and 8 mg/L nitrite nitrogen for 30 days, measuring survival, growth performance, hepatopancreas enzyme activities (ACP, AKP, T-AOC, T-SOD), and conducting transcriptomic profiling of gill and hepatopancreas tissues. Chronic nitrite exposure significantly reduced weight gain rate from 82.98% in the control to 39.33% (4 mg/L) and 33.86% (8 mg/L), with survival declining from 58.89% to 51.11% and 38.89%, respectively. ACP activity and antioxidant capacities (T-AOC and T-SOD) were markedly suppressed, indicating impaired immune and oxidative stress defenses. Transcriptomic analysis revealed tissue-specific responses: gills primarily adjusted nitrogen/amino-acid metabolism at 4 mg/L and mitochondrial oxidative phosphorylation at 8 mg/L, whereas the hepatopancreas showed broader reprogramming involving cell junction integrity, tryptophan–kynurenine metabolism, cofactor/vitamin metabolism, lipid remodeling, cytochrome P450-mediated detoxification, and melanization-related immune pathways at higher stress. These findings demonstrate that chronic nitrite stress progressively erodes shrimp homeostasis through tissue-specific transcriptional reprogramming, forcing resource allocation toward maintenance and defense at the expense of growth, thereby providing a mechanistic basis for nitrite risk assessment and threshold management in aquaculture.

## Simple Summary

Nitrite is a hidden threat in shrimp ponds: it can harm shrimp even when concentrations are not high enough to kill them outright. To understand the long-term effects of this quiet stress, we cultured black tiger shrimp (*Penaeus monodon*) for 30 days in water with low to moderate nitrite levels (4 and 8 mg/L). Compared with shrimp in clean water, those exposed to nitrite grew more slowly and had lower survival. Their immune defenses and ability to cope with oxidative stress were also weakened. By examining gene expression in two key tissues (gills and hepatopancreas), the study revealed that each tissue responds differently. Gills mainly adjust how they handle nitrogen and produce energy, while the hepatopancreas turns on a wider set of protective responses, including detoxification, immune defense, and vitamin-related metabolism. These findings help explain why shrimp in nitrite-polluted water often perform poorly over time, even without obvious signs of poisoning. The results can help farmers recognize hidden nitrite damage early and guide safer water quality limits to reduce losses.:

## 1. Introduction

*Penaeus monodon* is one of the most important farmed shrimp species in tropical and subtropical regions and is widely used as a model organism for investigating environmental adaptation and health homeostasis in crustaceans [1]. As shrimp aquaculture increasingly shifts toward high-density and intensive production, nitrogen and organic loads tend to accumulate more readily, making water-quality fluctuations a growing constraint on shrimp growth and health. Among nitrogenous pollutants, nitrite nitrogen is a common and representative toxic intermediate in aquaculture systems [2]. Its concentration is jointly shaped by feeding intensity, decomposition of uneaten feed and feces, dissolved oxygen availability, nitrification efficiency of microbial communities, and water-column stratification, resulting in pronounced variability and episodic elevation. Notably, nitrite nitrogen often persists at low-to-moderate levels in production settings, where it may cause substantial but difficult-to-detect “hidden” losses without obvious external symptoms. Clarifying the long-term effects of nitrite exposure and identifying the key pathways through which it impairs shrimp performance are therefore of considerable scientific and practical importance [3].

Compared with acute toxicity incidents, nitrite more commonly affects cultured organisms through chronic, sublethal exposure. Under such conditions, shrimp may maintain relatively high survival, yet physiological homeostasis can be persistently disrupted, ultimately manifesting as reduced growth rate, poorer feed-use efficiency, and weakened stress tolerance [4,5]. Mechanistically, nitrite may interfere with respiration and ion regulation, provoke oxidative stress, and compromise the function of key metabolic organs, thereby shifting energy allocation and altering immune effectiveness [6,7,8]. These changes are often cumulative and insidious, and they can overlap with the effects of other production variables such as stocking density, temperature, and feed quality, increasing uncertainty in risk attribution and management decisions. Establishing a quantitative evidence chain linking “chronic stress intensity–physiological responses–growth outcomes” under controlled exposure conditions is thus essential for explaining nitrite-associated performance fluctuations in shrimp culture [9].

From a mechanistic perspective, transcriptomic responses provide a key molecular interface linking external environmental stressors to host health and growth phenotypes [10]. Environmental challenges often first trigger tissue-level reprogramming of gene expression, which then coordinates changes in antioxidant defense, inflammatory and immune regulation, detoxification and transport, energy allocation, and ion and osmotic homeostasis [11,12,13]. In *P. monodon*, the gills are directly exposed to the rearing water and serve as the frontline for ion exchange and gas exchange, typically exhibiting rapid and sensitive stress signals [14]. In contrast, the hepatopancreas functions as a metabolic hub with detoxification and immune roles, and therefore better reflects compensatory regulation and energy reallocation under chronic stress [15]. However, current studies still rarely integrate growth outcomes, physiological and biochemical phenotypes, and whole-transcriptome changes in gill and hepatopancreas within the same chronic exposure framework, limiting the systematic identification of key pathways and hub signals [16].

To address this gap, we exposed *P. monodon* to chronic nitrite nitrogen (NO_2_^−^-N, mg·L^−1^) at 0, 4, and 8 mg·L^−1^, continuously monitoring survival and growth-related production traits while tracking oxidative stress and immune/metabolic status using physiological and biochemical indices. At the molecular level, gill and hepatopancreas tissues were collected for RNA sequencing, and differential expression together with functional and pathway enrichment analyses were used to pinpoint biological processes and putative metabolic hub genes that covary with stress intensity and phenotypic shifts. By comparing tissue-specific division of labor and coordination patterns and further integrating physiological indices with transcriptomic signals, we propose an explanatory framework linking chronic stress intensity to tissue-specific transcriptional regulation, homeostatic remodeling, and ultimately altered growth performance. This work provides a more quantitative mechanistic basis for nitrite risk assessment and threshold management in aquaculture practice.

## 2. Materials and Methods

### 2.1. Study Site and Experimental Animals

The experiment was conducted at the Shenzhen Base of the South China Sea Fisheries Research Institute, Chinese Academy of Fishery Sciences (CAFS). A total of 270 healthy, size-uniform *P. monodon* were randomly selected, with an initial body weight of approximately 2.2 ± 0.14 g. Shrimp were transferred from the culture pond to 200-L white plastic tanks and acclimated for 3 days to minimize handling and transfer stress prior to the exposure trial. During acclimation, natural seawater was allowed to settle and was thoroughly aerated before use. Throughout the experiment, salinity was maintained at 28–30‰, temperature at 26–30 °C and pH at 8.0–8.2; dissolved oxygen was kept at 6.5–7.5 mg/L using continuous micro-aeration to ensure consistent water conditions. Total ammonia nitrogen (TAN) and nitrate nitrogen (NO_3_^−^-N) remained below 0.02 mg/L in all treatments throughout the experiment, with no significant differences among groups. Shrimp were fed three times daily with a formulated grass shrimp diet (Dongteng Feed Co., Ltd., Zhanjiang, China) at a daily ration of 5% of body weight; the diet contained the following proximate composition (according to the manufacturer’s specification): crude protein ≥ 42%, crude lipid ≥ 4%, crude fiber ≤ 5%, crude ash ≤ 16%, moisture ≤ 12%, and total phosphorus 0.60–1.60%. One hour after each feeding, uneaten feed and feces were removed by siphoning. Shrimp were fasted for 24 h before the start of the exposure experiment.

### 2.2. Experimental Design for Chronic Nitrite-Nitrogen Exposure

Based on previous findings [5,17], 4 mg/L NO_2_^−^-N was selected as a relatively low and comparatively safe exposure concentration, expected to allow shrimp to maintain relatively normal growth and survival, while 8 mg/L NO_2_^−^-N was chosen as a concentration at which nitrite-related adverse effects could potentially occur. A single-factor design was therefore applied with three treatments: a control group (source seawater after filtration and aeration, 0.017 mg/L NO_2_^−^-N, designated N0.) and two NO_2_^−^-N exposure groups (4 mg/L and 8 mg/L, designated N4 and N8, respectively). Each treatment included three replicates, with 30 shrimp per replicate. Analytical-grade sodium nitrite (NaNO_2_; Sinopharm Chemical Reagent Co., Ltd., Shanghai, China) was used to prepare and regulate nitrite-nitrogen levels in the water. The exposure period lasted 30 days. During the trial, water was renewed every 2 days by replacing half of the tank volume; NaNO_2_ was replenished immediately after water exchange in the exposure groups to restore target concentrations. Nitrite-nitrogen concentrations were measured daily using a spectrophotometric method and adjusted as needed to maintain stability across treatments.

### 2.3. Sample Collection and Storage

At the end of the experiment, all shrimp were fasted for 24 h. The number of surviving shrimp in each replicate tank was recorded, and individual body weights were measured for growth-performance calculations. Tissue sampling was conducted under aseptic conditions. For each replicate, shrimp were randomly selected and dissected. Gill and hepatopancreas tissues were excised, placed into sterile cryotubes, snap-frozen in liquid nitrogen, and stored at −80 °C, with tissue-specific samples prefixed as ‘G_’ for gill and ‘H_’ for hepatopancreas. In parallel, hepatopancreas tissues designated for physiological assays were collected into sterile cryotubes, snap-frozen in liquid nitrogen, and stored at −80 °C for enzyme activity and antioxidant measurements.

### 2.4. Growth Performance Indices

Survival rate (SR) and weight gain rate (WGR) were calculated as follows:

SR (%) = Nt/N0 × 100

WGR (%) = (Wt − W0)/W0 × 100

where N0 is the initial number of shrimp, Nt is the final number of shrimp, W0 is the initial mean body weight (g), and Wt is the final mean body weight (g).

### 2.5. Hepatopancreas Enzyme Activities and Antioxidant Indices

Hepatopancreas samples were thawed on ice and weighed. Tissues were homogenized in pre-chilled PBS (1:9, *w*/*v*; Thermo Fisher Scientific, Waltham, MA, USA) under ice-bath conditions. Homogenates were centrifuged at 2500× *g* for 10 min, and the supernatant was collected as the test sample (10% tissue homogenate). Commercial assay kits were used to determine alkaline phosphatase (AKP), acid phosphatase (ACP), total antioxidant capacity (T-AOC), and total superoxide dismutase (T-SOD), following the manufacturers’ instructions [18]. To minimize degradation, sample preparation and assays were performed at low temperature and completed as rapidly as possible.

### 2.6. Transcriptome Sequencing and Bioinformatic Analysis

For transcriptomic analysis, one shrimp was randomly selected from each replicate tank, and its gill and hepatopancreas tissues were dissected and processed individually. Thus, a total of 9 individuals (3 treatments × 3 replicates) were subjected to RNA-seq, with each replicate tank representing an independent biological replicate (*n* = 3 per treatment). Total RNA was extracted from each sample and assessed for integrity and purity, and qualified RNA was used for cDNA library construction. Library preparation and sequencing were performed by a commercial service provider using an Illumina high-throughput sequencing platform to generate raw reads.

Raw data were subjected to quality control, including adaptor trimming, removal of low-quality reads, and filtering of overly short reads, to obtain clean reads [19]. Clean reads were then aligned to the *P. monodon* reference genome for expression quantification, and gene expression levels were calculated [20,21]. Differential expression analyses were conducted with DESeq2 package v1.50.2 to identify differentially expressed genes (DEGs) [22]. GO and KEGG enrichment analyses were subsequently performed on DEGs to infer biological processes and regulatory pathways associated with stress intensity [23,24]. In addition, clustering analysis and principal component analysis (PCA) were used to compare global expression profiles across treatments and tissues [25].

### 2.7. Protein–Protein Interaction (PPI) Network Analysis

To explore potential functional interactions between DEGs obtained from different tissues, we performed PPI network analysis using the STRING database (v12.0), which combines known and predicted protein–protein associations. The networks were constructed with active interaction sources (text mining, experiments, databases, and co-expression), species limitation to “*Penaeus vannamei*”, and a minimum interaction score of 0.4. Following the acquisition of the protein–protein interaction network, NetworkX in Python (v3.6.1) was employed to visualize the interaction network of the target genes.

### 2.8. Quantitative Real-Time (qRT) PCR Validation of DEGs

To verify the reliability of the RNA-seq data, ten candidate genes were randomly selected for qRT-PCR analysis. Gene-specific primers were designed using Primer Premier v5.0 software. In this research, EF-1α was employed as the internal control. Total RNA was isolated from hepatopancreas and gill tissues using TRIzol reagent (Invitrogen, Carlsbad, CA, USA), and cDNA was synthesized with the Evo M-MLV Reverse Transcription Kit (Accurate Biotechnology Co., Ltd., Guangzhou, China). qRT-PCR was conducted on a Roche LightCycler^®^ 480II Real-Time PCR System (Roche, Basel, Switzerland), with three technical replicates performed for each sample and the internal control.

### 2.9. Statistical Analysis and Visualization

Continuous variables are presented as mean ± standard deviation (mean ± SD). For percentage data, SR was arcsine square-root transformed prior to analysis, whereas WGR was analyzed using the original values. Normality and homogeneity of variance were assessed using the Shapiro–Wilk test and Levene’s test, respectively. When assumptions were satisfied, one-way analysis of variance (ANOVA) was used to compare treatment groups, followed by Tukey’s honestly significant difference (HSD) test for pairwise comparisons. When assumptions were not met, data were log-transformed or analyzed using appropriate non-parametric tests. All tests were two-sided, with *p* < 0.05 considered statistically significant. Significant differences are denoted by different letters.

For transcriptomic data, differential expression analysis was conducted between each nitrite-exposed group and the corresponding control within the same tissue, and multiple testing was controlled using the false discovery rate (FDR); adjusted *p* values (Padj) are reported. Functional enrichment analyses (GO and KEGG) were performed on differentially expressed genes with FDR correction. Principal component analysis (PCA), volcano plots, and enrichment bubble plots were generated to visualize global expression patterns and enrichment results.

## 3. Results

### 3.1. Changes in Survival and Growth Performance of P. monodon

To evaluate the effects of different nitrite-nitrogen exposure levels on production performance in *P. monodon*, survival rate (SR), initial mean body weight (W0), final mean body weight (Wt), and weight gain rate (WGR) were compared among groups (Table 1). At the beginning of the experiment, W0 ranged from 2.17 to 2.28 g with no significant differences among treatments, indicating comparable starting conditions. At the end of the trial, SR in the control group was 58.89 ± 9.58%, which was significantly higher than that in the 8 mg/L group (38.89 ± 7.53%, *p* < 0.05). The 4 mg/L group showed an intermediate SR (51.11 ± 9.82%). Consistent with this pattern, Wt was significantly reduced in the exposure groups: the control group reached 3.97 ± 0.24 g, whereas the 8 mg/L groups reached 2.95 ± 0.16 g, respectively (*p* < 0.05), indicating suppressed biomass accumulation under nitrite exposure. WGR further reflected a marked reduction in growth efficiency, decreasing from 82.98 ± 11.40% in the control to 39.33 ± 9.36% and 33.86 ± 6.16% in the 4 mg/L and 8 mg/L groups, respectively (*p* < 0.05). Collectively, these findings demonstrate that exposure to nitrite nitrogen at 4–8 mg/L significantly reduced final body weight and weight gain rate and was accompanied by a declining trend in survival.

### 3.2. Responses of Enzyme Activities and Antioxidant Capacity in P. monodon

To further characterize physiological responses to nitrite exposure, hepatopancreas phosphatase activities (ACP and AKP) and antioxidant indices (T-AOC and T-SOD) were determined. Acid phosphatase (ACP) activity was significantly reduced in the exposure groups: ACP was 0.25 ± 0.03 U/mgprot in the control, but decreased to 0.13 ± 0.02 U/mgprot and 0.16 ± 0.02 U/mgprot in the 4 mg/L and 8 mg/L groups, respectively (*p* < 0.05; Table 2). In contrast, alkaline phosphatase (AKP) did not differ significantly among groups, suggesting a weaker or more variable AKP response under the present conditions (Table 2).

T-AOC tended to decrease with increasing nitrite concentration and was significantly lower in the 8 mg/L group (0.83 ± 0.16 U/mgprot) than in the control (1.65 ± 0.70 U/mgprot) (*p* < 0.05; Table 3). T-SOD showed a similar pattern: no significant difference was detected between the control (142.14 ± 31.48 U/mgprot) and the 4 mg/L group (104.62 ± 25.60 U/mgprot), whereas T-SOD was significantly reduced in the 8 mg/L group (46.10 ± 34.81 U/mgprot) compared with the control (*p* < 0.05; Table 3). Taken together, these results indicate that nitrite exposure significantly suppressed ACP activity and, at the higher exposure level, markedly reduced antioxidant capacity.

### 3.3. Global Transcriptomic Separation and Differential Expression in Gill and Hepatopancreas

**Figure 1:**
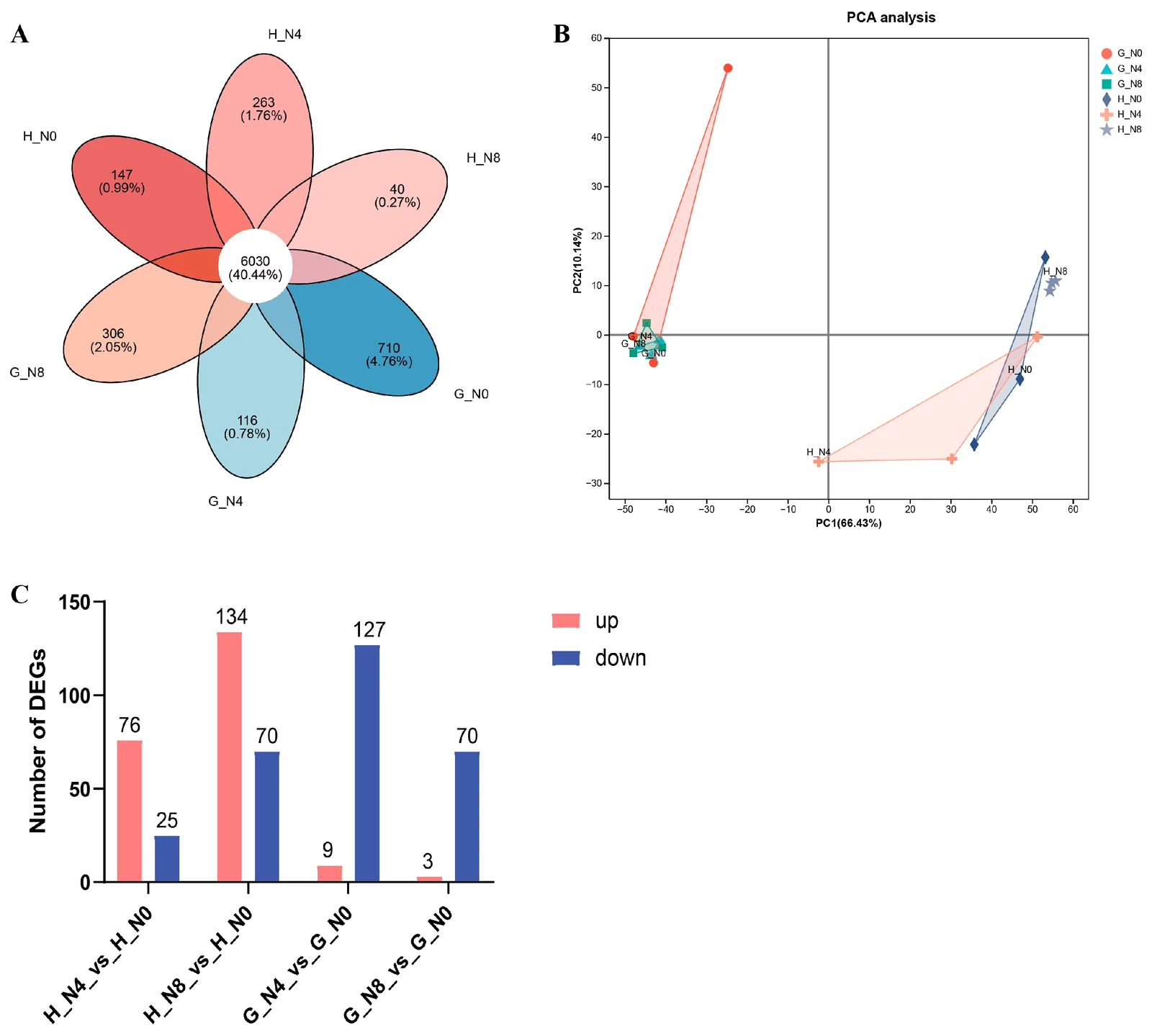
Overview of transcriptomic differences in gill and hepatopancreas of *P. monodon* under chronic nitrite nitrogen exposure. (**A**) Shared and group-specific expressed genes among groups (Venn diagram). (**B**) Principal component analysis (PCA). (**C**) Number of differentially expressed genes.

We compared gill (G) and hepatopancreas (H) transcriptomes among the three NO_2_^−^-N treatments. A total of 6030 genes (40.44%) were shared across all six groups (Figure 1A). Group-specific genes differed by tissue and exposure level. In gills, the largest number of unique genes was observed in G_N0 (710; 4.76%), followed by G_N8 (306; 2.05%) and G_N4 (116; 0.78%). In the hepatopancreas, H_N4 had the most unique genes (263; 1.76%), followed by H_N0 (147; 0.99%) and H_N8 (40; 0.27%).

Principal component analysis (PCA) showed that inter-sample variation was dominated by tissue origin. PC1 explained 66.43% of the total variance and PC2 explained 10.14% (Figure 1B). Gill samples clustered on the negative side of PC1, whereas hepatopancreas samples clustered on the positive side, indicating clear baseline separation between the two tissues (Figure 1B). Within each tissue, samples also displayed partial separation across exposure levels.

DEG analysis further revealed tissue-specific response patterns (Figure 1C). In gills, G_N4 vs. G_N0 identified 9 upregulated and 127 downregulated genes, while G_N8 vs. G_N0 identified 5 upregulated and 70 downregulated genes, indicating a predominantly downregulated response that was less pronounced at the higher concentration. In the hepatopancreas, H_N4 vs. H_N0 identified 76 upregulated and 25 downregulated genes, and H_N8 vs. H_N0 identified 134 upregulated and 70 downregulated genes, indicating a stronger and more activation-dominated response that increased with exposure concentration.

### 3.4. Differential Expression of Candidate Genes in Gill and Hepatopancreas

**Figure 2:**
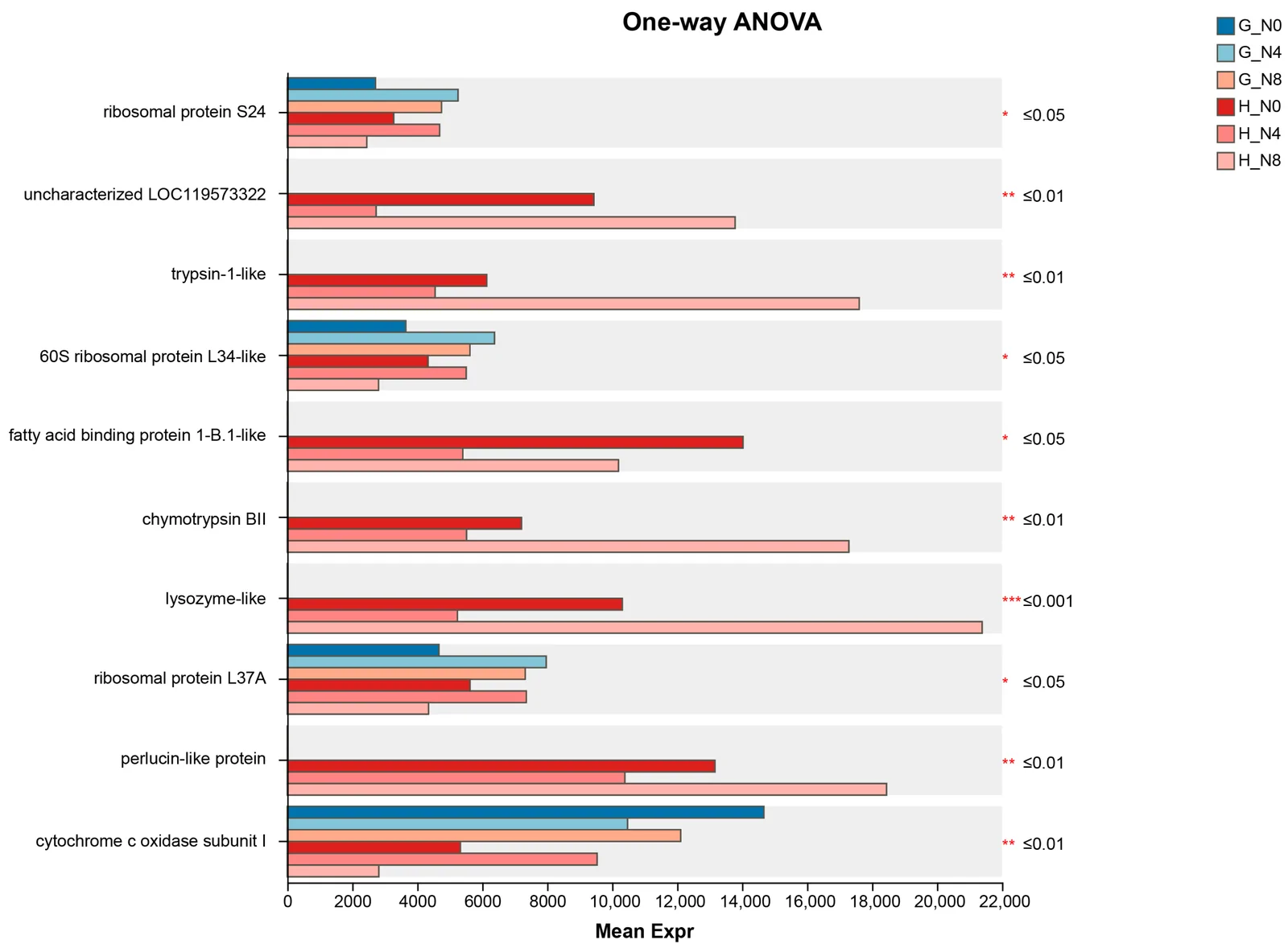
Mean expression of candidate genes in gills (G) and hepatopancreas (H) across nitrite nitrogen treatments with one-way ANOVA. The *x*-axis shows mean expression (Mean Expr) and the *y*-axis lists genes. Colors indicate groups. Asterisks denote significance (* *p* ≤ 0.05, ** *p* ≤ 0.01, *** *p* ≤ 0.001).

To validate the expression responses of key candidate genes across tissues and nitrite concentrations, we performed one-way ANOVA on gene expression levels in gill (G) and hepatopancreas (H) across the six groups (N0/N4/N8) (Figure 2). Results showed that all 10 candidate genes differed significantly among groups (*p* < 0.05; Figure 2). In the hepatopancreas, several digestive- and immune-related genes, including lysozyme-like, trypsin-1-like, chymotrypsin BII, perlucin-like, and the uncharacterized gene LOC119573322, were strongly induced and generally reached their highest expression at 8 mg/L. In contrast, the lipid transport-related gene fatty acid binding protein 1-B.1-like (LOC119581179) was highest in the control and decreased under nitrite exposure, with partial recovery at 8 mg/L. Tissue differences were also evident for energy metabolism and translation-related genes: cytochrome c oxidase subunit I (COX1) was generally higher in gills but lower in the hepatopancreas at 8 mg/L, whereas ribosomal protein genes (L37A, 60S L34-like, and S24) were relatively higher in gills and often peaked at 4 mg/L.

### 3.5. GO and KEGG Enrichment of Differentially Expressed Genes in Gills

**Figure 3:**
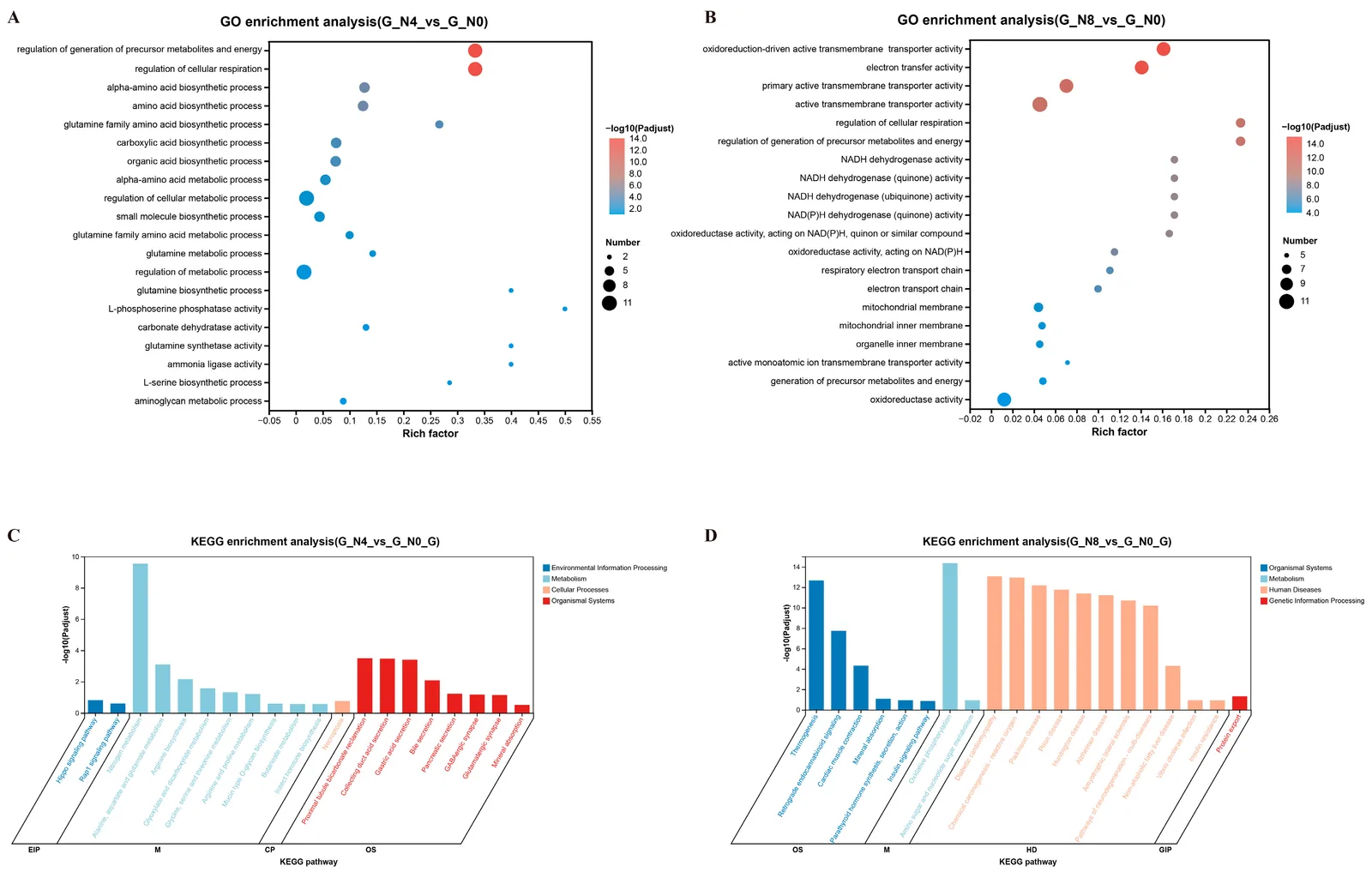
GO and KEGG enrichment analyses of DEGs in gills (G). (**A**) GO enrichment bubble plot for G_N4 vs. G_N0. (**B**) GO enrichment bubble plot for G_N8 vs. G_N0. (**C**) KEGG enrichment for G_N4 vs. G_N0. (**D**) KEGG enrichment for G_N8 vs. G_N0. In bubble plots, the *x*-axis represents the rich factor, bubble size indicates the number of enriched genes, and color denotes significance (−log10 Padj). N0, N4, and N8 represent 0, 4, and 8 mg/L nitrite nitrogen, respectively.

To elucidate the key molecular processes altered in gills (G) after chronic nitrite nitrogen exposure, we performed GO and KEGG enrichment analyses for DEGs identified in G_N4 vs. G_N0 and G_N8 vs. G_N0. Under 4 mg/L exposure (G_N4 vs. G_N0), GO enrichment was mainly associated with amino acid biosynthesis and metabolism, including α-amino acid and amino acid biosynthetic processes, glutamine-family amino-acid metabolism, and carboxylic/organic-acid and small-molecule biosynthesis (Figure 3A). Terms related to cellular respiration and precursor metabolite generation were also enriched, together with enzyme activities such as glutamine synthetase activity and carbonic anhydrase/carbonate dehydratase activity. KEGG analysis showed significant enrichment in nitrogen metabolism and multiple amino acid metabolic and biosynthetic pathways, including alanine–aspartate–glutamate metabolism, arginine biosynthesis, glycine–serine–threonine metabolism, arginine–proline metabolism, glyoxylate and dicarboxylate metabolism, and butanoate metabolism (Figure 3C). In addition, modules related to ion and acid–base homeostasis, such as bicarbonate transport and acid secretion, were enriched.

Under 8 mg/L exposure (G_N8 vs. G_N0), GO enrichment shifted toward transmembrane transport and redox-linked electron transfer, including redox-driven active transmembrane transport, electron-transfer activity, NADH/NAD(P)H dehydrogenase-related functions, respiratory and electron transport chains, and mitochondrial inner-membrane components (Figure 3B). KEGG results similarly showed enrichment of oxidative phosphorylation and thermogenesis/energy-metabolism regulation pathways, together with conserved modules related to protein homeostasis and cellular stress responses (Figure 3D).

### 3.6. GO and KEGG Enrichment of Differentially Expressed Genes in Hepatopancreas

**Figure 4:**
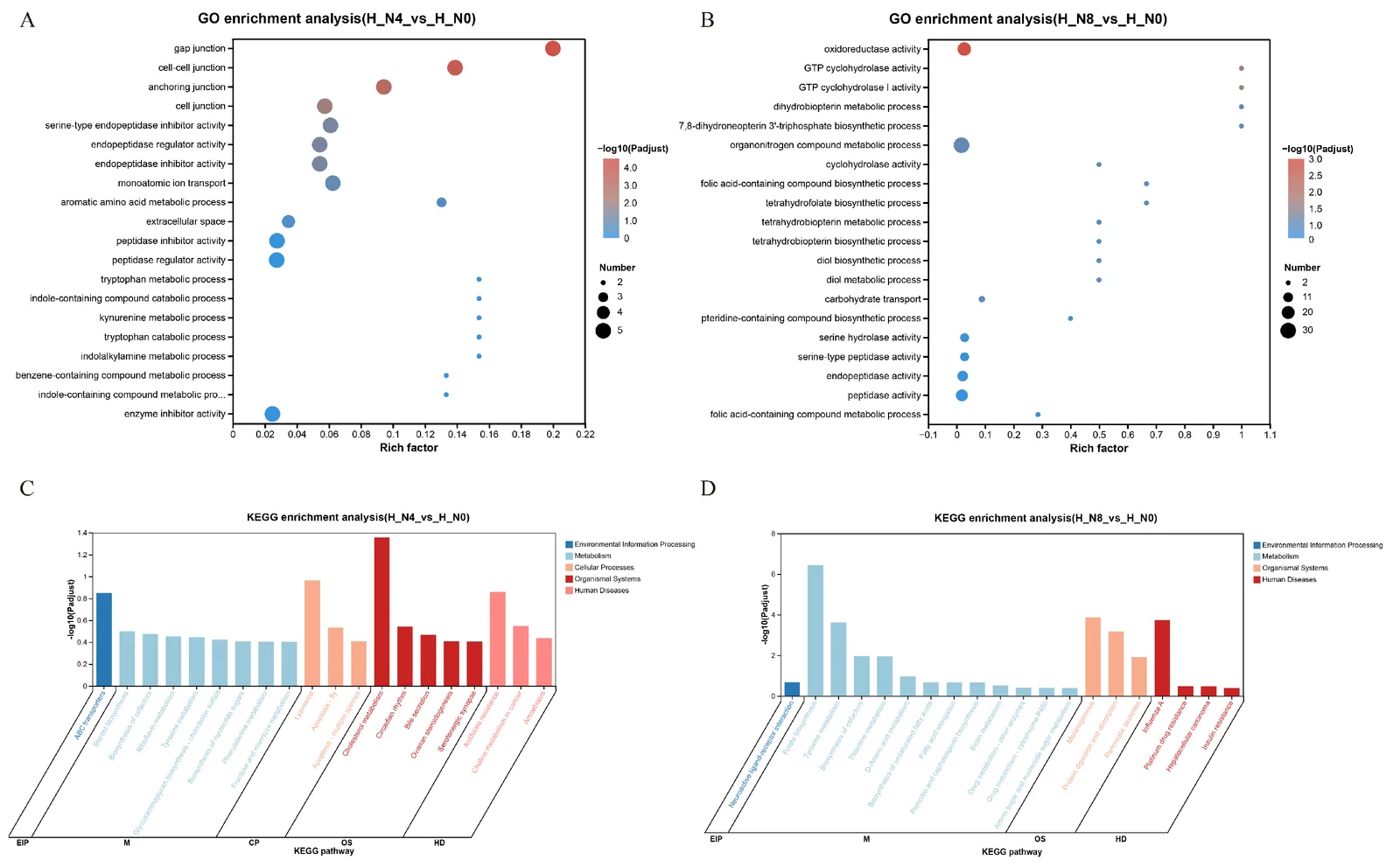
GO and KEGG enrichment analyses of DEGs in hepatopancreas (H). (**A**) GO enrichment bubble plot for H_N4 vs. H_N0. (**B**) GO enrichment bubble plot for H_N8 vs. H_N0. (**C**) KEGG enrichment for H_N4 vs. H_N0. (**D**) KEGG enrichment for H_N8 vs. H_N0. In bubble plots, the *x*-axis represents the rich factor, bubble size indicates the number of enriched genes, and color denotes significance (−log10 Padj). N0, N4, and N8 represent 0, 4, and 8 mg/L nitrite nitrogen, respectively.

To characterize molecular processes altered in the hepatopancreas (H) during chronic nitrite exposure, we conducted GO and KEGG enrichment analyses for DEGs from H_N4 vs. H_N0 and H_N8 vs. H_N0. Under 4 mg/L exposure (H_N4 vs. H_N0), GO enrichment primarily pointed to cell junction and barrier-associated processes, including gap junction, cell–cell junction, anchoring junction, and cell junction terms (Figure 4A). Protease inhibition and regulation terms were also enriched, such as serine-type endopeptidase inhibitor activity and endopeptidase/peptidase inhibitor and regulator activity, together with monoatomic ion transport, aromatic amino-acid metabolism, and the tryptophan–kynurenine branch. KEGG analysis showed enrichment in transmembrane transport and metabolic pathways, including ABC transporters, steroid/sterol-related metabolism, biosynthesis of cofactors, riboflavin metabolism, fructose and mannose metabolism, and nucleotide-sugar-related pathways (Figure 4C). Lysosome, apoptosis, circadian rhythm, and serotonin-related signaling modules were also enriched.

Under 8 mg/L exposure (H_N8 vs. H_N0), GO enrichment shifted toward redox processes and cofactor metabolism, including oxidoreductase activity, GTP cyclohydrolase-related functions, and terms linked to (tetrahydro)biopterin/pteridine and folate/tetrahydrofolate metabolism (Figure 4B). Biotin metabolism/biosynthesis, carbohydrate transport, and serine hydrolase/peptidase activities were also enriched. KEGG analysis further revealed pathways associated with vitamin and aromatic amino-acid metabolism, such as folate biosynthesis, thiamine/biotin metabolism, and tyrosine metabolism (Figure 4D). Lipid remodeling pathways, including biosynthesis of unsaturated fatty acids and fatty acid elongation, were enriched, together with detoxification-related modules involving cytochrome P450 and melanogenesis.

### 3.7. Interaction Networks of Key Modules and GSEA Validation

**Figure 5:**
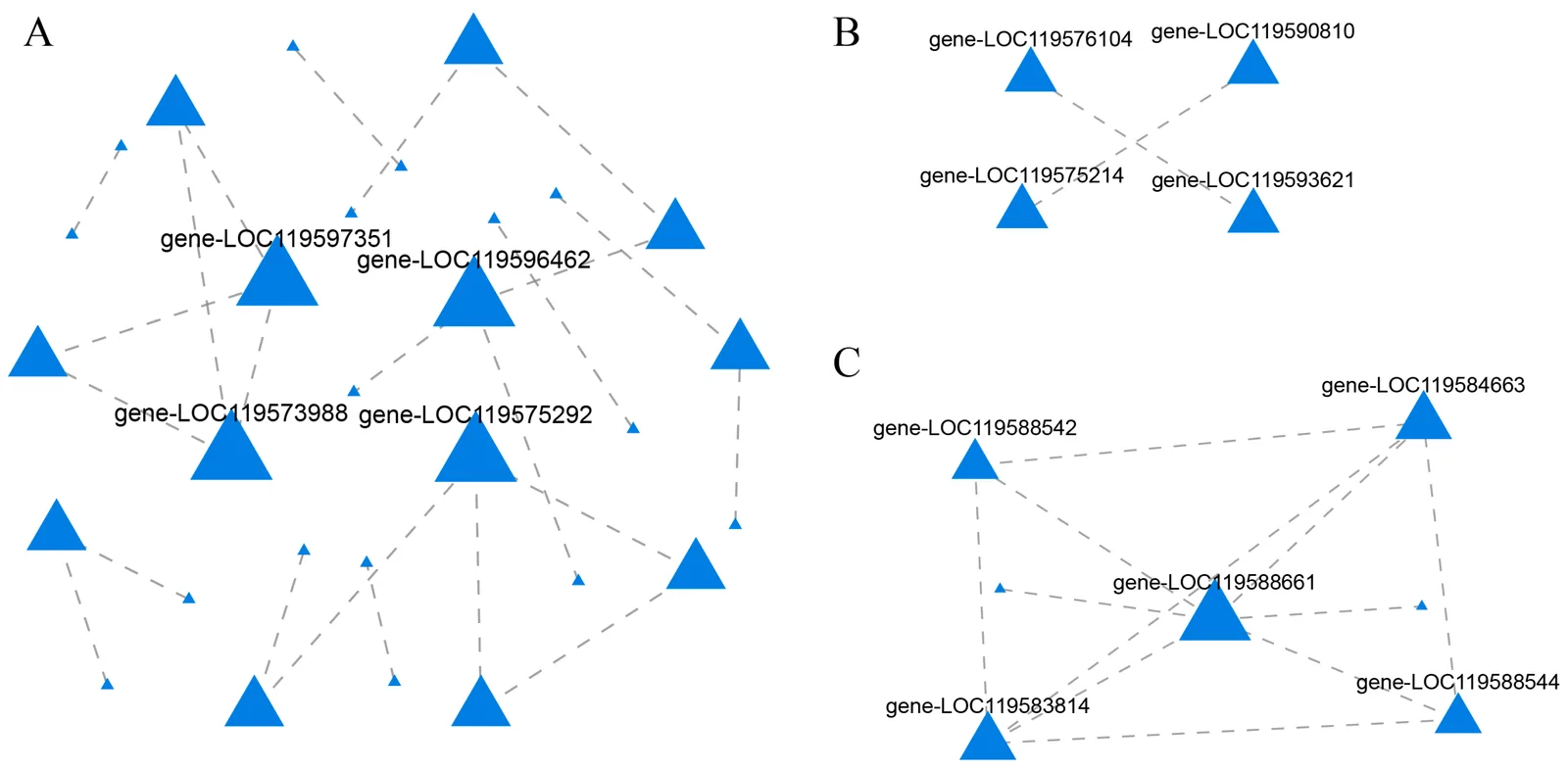
Interaction networks of DEGs. (**A**) Key-gene interaction network for hepatopancreas H_N8 vs. H_N0. (**B**) Key module for hepatopancreas H_N4 vs. H_N0. (**C**) Key module for gills G_N4 vs. G_N0.

To identify core regulatory modules under nitrite stress, PPI networks were constructed for DEGs from the key comparisons (Figure 5). In H_N8 vs. H_N0, the network was dominated by four highly connected hubs: probable U3 small nucleolar RNA-associated protein 11 (LOC119597351), GEM-associated protein 5-like (LOC119573988), S-adenosylmethionine synthase-like (LOC119596462), and 39S ribosomal protein L3 (LOC119575292) (Figure 5A). These hubs are associated with nucleolar/ribosome biogenesis and RNA processing, post-transcriptional regulation, methyl-donor metabolism via SAM synthesis, and mitochondrial ribosomal translation, respectively. In H_N4 vs. H_N0, the network was more concentrated on endocrine/nuclear receptor regulation and aromatic amino acid catabolism, with hubs including isatin hydrolase-like (LOC119576104), hormone receptor 4-like (LOC119590810), probable nuclear hormone receptor HR3 (LOC119575214), and kynureninase-like (LOC119593621) (Figure 5B). In the gill module (Figure 5C), enriched nodes were mainly related to nitrogen assimilation and amino-acid metabolism/transport, including glutamine synthetase (LOC119588542), glutamine synthetase-like (LOC119588544), glutamate synthase [NADH] (LOC119584663), delta-1-pyrroline-5-carboxylate synthase-like (LOC119588661), and putative ammonium transporter 1 (LOC119583814).

**Figure 6:**
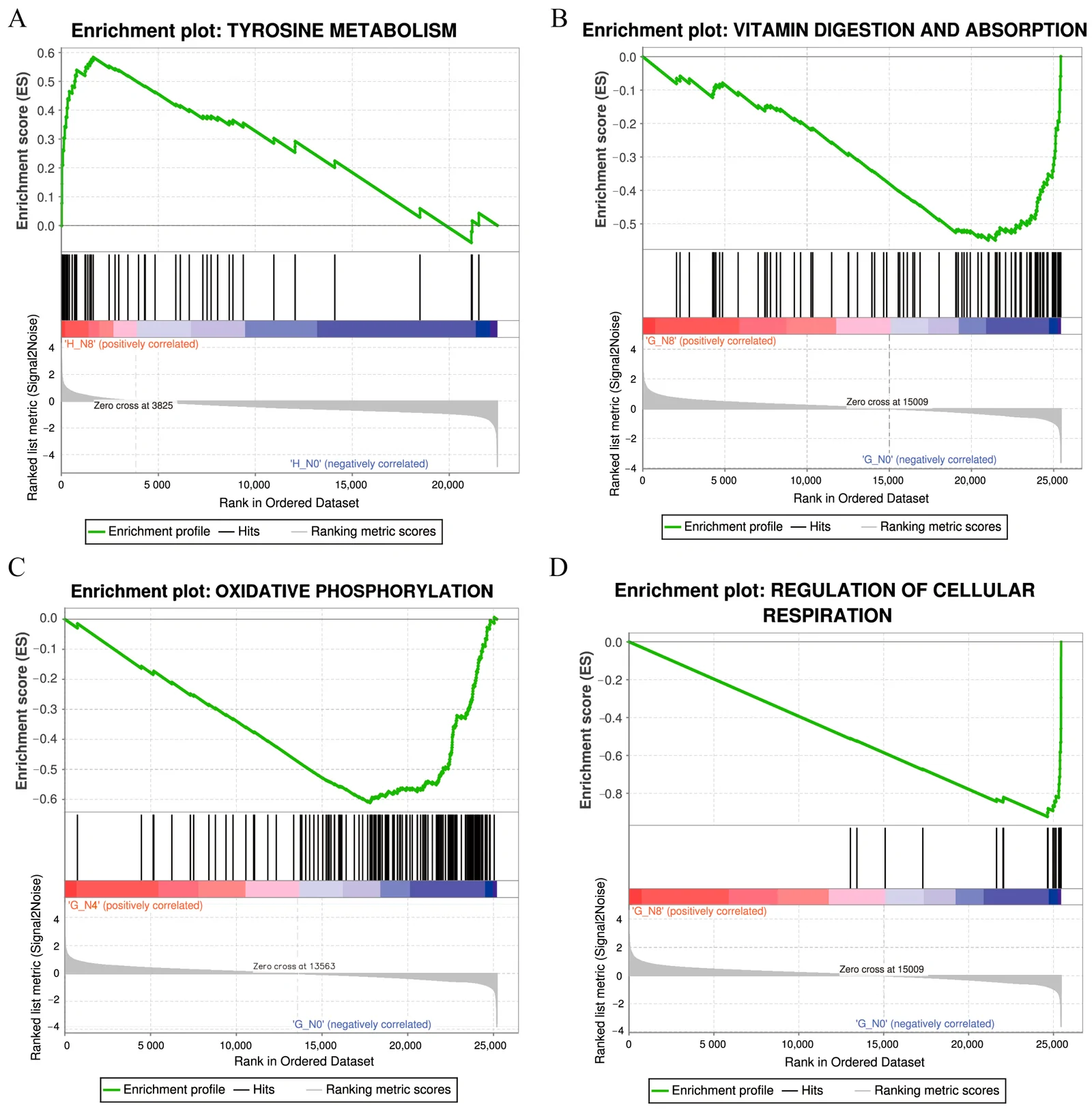
Representative GSEA enrichment curves. (**A**) Tyrosine metabolism. (**B**) Vitamin digestion and absorption. (**C**) Oxidative phosphorylation. (**D**) Regulation of cellular respiration. The green curve indicates the enrichment score (ES), black vertical bars mark gene-set hits, and the bottom color strip shows the direction of association with the ranked gene list. N0, N4, and N8 represent 0, 4, and 8 mg/L nitrite nitrogen, respectively.

GSEA enrichment curves further supported these modular responses (Figure 6). In H_N8 vs. H_N0, Tyrosine metabolism showed a pronounced enrichment signal, indicating system-wide engagement of aromatic amino-acid metabolism/catabolism under high-intensity stress (Figure 6A). In gill comparisons, gene sets related to Oxidative phosphorylation and Regulation of cellular respiration displayed clear enrichment differences (Figure 6C,D), consistent with remodeling of mitochondrial energy production and respiratory-chain processes under stress. In addition, the gene set for Vitamin digestion and absorption was also enriched (Figure 6B), suggesting altered pathways linked to vitamin and nutrient handling during chronic nitrite exposure.

### 3.8. Quantitative Real-Time PCR Validation of DEGs

**Figure 7:**
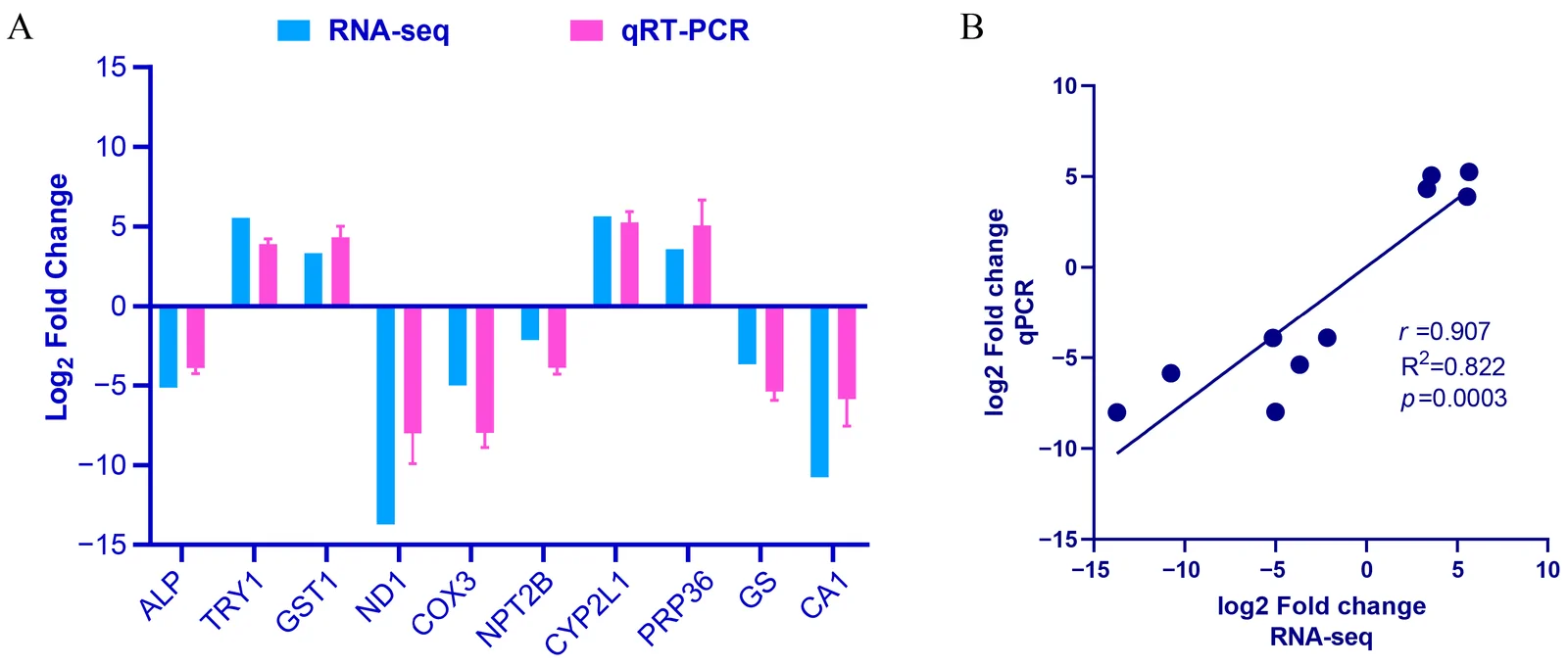
qPCR validation of DEGs. (**A**) Relative expression profiling of ten DEGs via qRT-PCR. (**B**) Regression analysis between qPCR and RNA-seq data.

To assess the reliability of mRNA-seq data, ten DEGs were randomly selected for qRT-PCR validation. The selected DEGs comprised four up-regulated genes (*trypsin-1*, *TRY1*; *glutathione S-transferase 1*, *GST1*; *proline-rich protein 36*, *PRP36*; and *cytochrome P450 2L1*, *CYP2L1*) and six down-regulated genes (*alkaline phosphatase*, *ALP*; *cytochrome c oxidase subunit III*, *COX3*; *NADH dehydrogenase subunit 1*, *ND1*; *sodium-dependent phosphate transport protein 2B*, *NPT2B*; *glutamine synthetase*, *GS*; and *carbonic anhydrase 1*, *CA1*). The expression trends determined by qRT-PCR were found to be consistent with those from the sequencing analysis (Figure 7). Pearson’s correlation analysis between RNA-Seq and qRT-PCR log_2_ fold changes revealed a strong and significant correlation (*r* = 0.907, *R*^2^ = 0.822, *p* < 0.001, *n* = 10), confirming the reliability of the RNA-Seq data.

## 4. Discussion

### 4.1. Dose-Dependent Growth Inhibition and Survival Decline Under Chronic Nitrite Exposure

The present study shows that, under comparable initial body weights, chronic exposure to nitrite nitrogen at 4–8 mg/L imposed a clear growth cost in *P. monodon*: weight gain rate decreased from 82.98% in the control to 39.33% and 33.86% at 4 and 8 mg/L, respectively, while survival declined from 58.89% to 51.11% and 38.89%. At N4, WGR was already sharply reduced, whereas SR remained statistically comparable to the control, indicating that growth suppression precedes mortality. At N8, SR decreased significantly, suggesting that compensatory capacity was gradually exhausted. This “growth-first, survival-later” pattern aligns well with the chronic-risk scenario commonly observed in intensive aquaculture, where the dominant outcome is not acute mass mortality but gradual erosion of performance, ultimately leading to systematic reductions in yield and market size. Chen et al. [26] reported that key phenotypes (growth and molting frequency) can shift within a 2–20 mg/L nitrite range, with 4 and 8 mg/L falling within a concentration window that produces clear biological effects, and review evidence has similarly emphasized that long-term exposure at 4–8 mg/L is sufficient to depress growth [27].

While the above results demonstrate a clear toxic effect of chronic nitrite exposure, the relatively low weight gain rate (82.98%) and survival (58.89%) in the control group need further consideration. This may be largely attributed to the relatively high stocking density employed in the experimental system. Specifically, 30 shrimp were reared in 200 L of water, corresponding to a stocking density of 150 ind/m^3^. This density is considerably higher than that typically used in commercial pond culture. Elevated stocking density is known to increase competition for food and space, elevate cannibalism, and impair growth and survival in penaeid shrimp. In a previous study, *P. monodon* juveniles reared at 150 ind/m^3^ exhibited similarly low growth and survival performance to those observed in the present control group, confirming that the baseline performance in this study is within the expected range under this specific density [18].

An additional factor that may have contributed to the relatively low control performance is the physiological status of the parental broodstock. In penaeid shrimp, prolonged captive reproduction can reduce egg quality: Anand et al. [28] reported that egg production and hatchability were markedly lower in first- and second-generation captive-reared *Penaeus indicus* than in wild broodstock. Maternal effects also strongly influence early juvenile growth. Benzie et al. [29] estimated that the dam component for total length and wet weight in *P. monodon* declined from approximately 0.5–0.6 at 6 weeks to 0.3–0.4 at 10 weeks, indicating that maternal influences are strongest early in development and contribute substantially to growth variation among families. The lower baseline growth and survival in the control group may have resulted from a late reproductive stage or variable egg quality of the broodstock, which remains a further investigation.

It should be emphasized that all treatments in the present study were maintained at identical stocking density and under the same environmental conditions. Therefore, any density-induced background stress was evenly distributed across groups and did not confound the comparison of nitrite effects. Linking dose to phenotype, the N4 group already diverted energy away from biomass accumulation, while at N8 the compensatory capacity was exhausted, resulting in simultaneous growth and survival penalties.

### 4.2. Reduced ACP and Declining Antioxidant Capacity Indicate Compromised Defensive Homeostasis

The hepatopancreas is a central organ for digestion, detoxification, and immune responses in *P. monodon* [30]. In this study, ACP was significantly lower in both exposure groups relative to the control, whereas AKP did not change significantly. This pattern suggests that chronic nitrite exposure may preferentially impair immune/phagolysosomal processes linked to acidic lysosomal pathways. In contrast, the non-significant AKP response may reflect tissue specificity, temporal dynamics of regulation, or higher inter-individual variability under the present conditions [31,32]. Differential sensitivity among immune-related enzymes under stress is frequently reported in shrimp studies, where ACP, AKP, T-AOC, and related indices are commonly used to characterize immune and antioxidant status and often change directionally with stress intensity [33,34].

More consistent evidence of impairment is provided by the antioxidant system. Here, T-AOC showed an overall downward trend, and T-SOD decreased significantly under the higher-intensity treatment, indicating consumption of antioxidant defenses and, in severe conditions, a state of “insufficient protection.” The significant suppression of T-AOC and T-SOD at 8 mg/L coincided with the lowest survival rate, suggesting that compromised antioxidant defenses contributed to the dose-dependent survival decline. In *Litopenaeus vannamei*, integrated histological–physiological–transcriptomic studies have shown that nitrite exposure can damage the hepatopancreas and gills and significantly reduce antioxidant indices such as T-AOC, SOD, and CAT [6]. When interpreted alongside the pronounced growth penalty observed here, a coherent explanation emerges: under prolonged nitrite stress, *P. monodon* must continuously allocate resources to sustain antioxidant and immune-related functions. When these defenses cannot fully offset damage, homeostasis is maintained in a costly manner that involves reduced growth efficiency and elevated maintenance demands, which ultimately results in substantial performance loss [35].

### 4.3. Divergent Transcriptional Strategies of Gills and Hepatopancreas Under Chronic Nitrite Stress

Our PCA provides a clear first conclusion: baseline transcriptomic differences between gills and hepatopancreas far exceeded treatment effects (PC1 = 66.43%), indicating that these two tissues are inherently highly specialized in their homeostatic functions, and nitrite exposure superimposes a secondary layer of remodeling on this tissue “baseline.” The magnitude and direction of differential expression further reinforce this division. Similar tissue-specific transcriptomic divergence has been observed in rainbow trout, where PCA of gene expression data from pyloric caecum, liver, and muscle revealed a clear separation among the three tissues, further supporting the notion that different tissues exhibit distinct transcriptional response patterns to physiological or environmental stimuli [36]. In gills, 4 mg/L yielded 9 upregulated and 127 downregulated DEGs, whereas 8 mg/L produced only 5 upregulated and 70 downregulated DEGs, reflecting a “convergent, predominantly downregulatory” response. The predominance of downregulated genes suggests a conservative transcriptional strategy under stress, wherein the gill suppresses energetically costly processes to conserve energy and limit damage, focusing limited transcriptional remodeling on maintaining core homeostatic functions. This interpretation aligns with their physiological roles: gills are the frontline interface with the water column, where sustained stimulation rapidly translates into ionic, acid–base, and respiratory burdens, favoring a transcriptomic profile that prioritizes maintenance over growth [37]. Moreover, the particularly modest number of DEGs at high concentration needs further consideration. One possible explanation is that the high-stress group exhibited greater inter-individual variability, arising from factors such as differential tissue damage, asynchronous molting, or variable tolerance. Such variability may have increased the within-group variance, consequently diminishing the statistical power to detect DEGs and possibly masking the full extent of transcriptional changes. In contrast, the hepatopancreas showed 76 upregulated and 25 downregulated DEGs at 4 mg/L, and a stronger reprogramming at 8 mg/L with 134 upregulated and 70 downregulated genes, characterized by a more pronounced activation-dominated pattern, consistent with its role as the hub for nutrient processing, energy allocation, detoxification, and immunity, which relies on active transcriptional regulation to compensate for stress-induced homeostatic perturbations [38]. Notably, the six groups still shared 6030 expressed genes (40.44%), suggesting that chronic exposure does not cause a wholesale “system collapse” in gene regulation, but rather tissue-specific rewiring and tuning of a common homeostatic network. This pattern is more consistent with the insidious, cumulative nature of chronic production losses observed in aquaculture.

### 4.4. Coordinated Nitrogen Assimilation, Mitochondrial Respiration, and Cofactor Metabolism Contribute to Elevated Maintenance Costs

By combining GO/KEGG enrichment with candidate genes and network modules, a more coherent mechanistic chain can be constructed. In gills, moderate stress (4 mg/L) primarily engaged nitrogen and amino acid metabolism (e.g., nitrogen metabolism, glutamate/glutamine-related pathways, arginine biosynthesis) together with terms linked to acid–base and ionic homeostasis. This matches the gill’s role as the frontline for osmoregulation and acid–base control and implies that stress adaptation is supported by redistribution of nitrogen-containing metabolic flux [35]. For instance, studies in rainbow trout have demonstrated the involvement of Rhesus (Rh) glycoproteins and H^+^-ATPase in branchial ammonia transport and excretion [39]. In addition, transcriptomic analyses in other species have revealed that ammonia stress modulates genes related to immune defense and cytoskeleton remodeling in gill tissue [40,41,42]. Network analysis further concretized this “nitrogen assimilation core” into a set of key nodes: putative ammonium transporter 1 (LOC119583814), glutamine synthetase/glutamine synthetase-like (LOC119588542/LOC119588544), glutamate synthase [NADH] (LOC119584663), and delta-1-pyrroline-5-carboxylate synthase-like (LOC119588661), which cover essential steps from ammonium entry and assimilation to nitrogen-skeleton redistribution and the proline/glutamate branch. Under stronger stress (8 mg/L), enrichment in gills shifted markedly toward mitochondrial inner-membrane functions, electron transport, NADH/NAD(P)H dehydrogenase activity, and redox-driven transmembrane transport, with GSEA corroborating signals for oxidative phosphorylation and regulation of cellular respiration. Similar metabolic shifts have been observed in other fish models under various stressors, where gill tissues prioritize pathways for energy production, such as glycolysis/gluconeogenesis and oxidative phosphorylation, to meet enhanced energetic demands [43,44]. This transition from nitrogen metabolic adjustment to respiratory-chain and energy-system remodeling was evidenced by the enrichment of oxidative phosphorylation and regulation of cellular respiration and by the qRT-PCR-validated downregulation of COX III and NADH dehydrogenase subunit 1.

The hepatopancreas followed a distinct but equally coherent trajectory. At 4 mg/L, enrichment emphasized cell junction and barrier-related processes (e.g., gap junction, cell junction) and regulation of proteolysis (multiple peptidase inhibitor/regulator categories), together with terms from the tryptophan–kynurenine branch, consistent with early adaptation at the level of “secretory and structural stability–proteolytic balance–aromatic amino-acid metabolism.” This coordinated engagement of junctional integrity and protease regulation aligns with the hepatopancreas’s frontline defensive role against environmental challenges [45]. At 8 mg/L, hepatopancreas enrichment shifted strongly toward oxidoreductase activities and a broad suite of cofactor/vitamin-related pathways (folate/tetrahydrofolate, biotin, thiamine, (tetrahydro)biopterin/pteridine compounds), accompanied by cytochrome P450–linked detoxification modules, lipid remodeling (unsaturated fatty acid biosynthesis and fatty acid elongation), and the melanization module associated with crustacean innate immunity. These transcriptional changes coincided with significant reductions in ACP, T-AOC, and T-SOD, together with the lowest WGR and SR. This dose-dependent coupling indicates that the expanded immune–detoxification–cofactor–lipid network in the hepatopancreas was accompanied by exhausted antioxidant defense and reduced growth and survival [46]. Candidate-gene patterns further ground the “hub burden” borne by the hepatopancreas in specific molecules: lysozyme-like, trypsin-1-like, chymotrypsin BII, and perlucin-like genes were markedly induced (especially at 8 mg/L), indicating strong engagement of immune effectors and digestive/secretory functions, whereas downregulation of a fatty acid binding protein 1-like gene suggests altered lipid transport and energy-substrate allocation [47]. Upstream network hubs converged on the same interpretation: S-adenosylmethionine synthase-like (LOC119596462) points to increased demand for methyl-donor supply and methylation-related metabolism, together with nucleolar/ribosome biogenesis and mitochondrial ribosomal components (probable U3 small nucleolar RNA-associated protein 11; 39S ribosomal protein L3) and a post-transcriptional regulatory node (GEM-associated protein 5-like), collectively indicating coordinated investment in protein synthesis/processing, mitochondrial function, and metabolic reprogramming [48]. Finally, GSEA signals for tyrosine metabolism and vitamin digestion/absorption provide additional support for aromatic amino-acid redistribution and adjustments in nutrient/cofactor provisioning [49]. Taken together, these lines of evidence converge on a central “chronic cost” of nitrite stress: the energetic demand for maintaining homeostasis, the need to reallocate nitrogen metabolism, and the requirement for sustained cofactor supply all rise, forcing resource reallocation toward survival and defense and ultimately manifesting as reduced growth efficiency and escalating survival pressure [50].

### 4.5. Integrated Dose-Dependent Metabolic Network and Maintenance-Cost Model Linking Key DEGs to Growth and Survivals

Integrating growth performance, physiological indices, and tissue-specific transcriptomic data, we propose a dose-dependent maintenance-cost model in which chronic nitrite nitrogen exposure forces *P. monodon* to reallocate resources from growth to homeostasis and defense. At 4 mg/L, the gill nitrogen-assimilation core—ammonium transporter 1, glutamine synthetase/glutamine synthetase-like, glutamate synthase [NADH], and delta-1-pyrroline-5-carboxylate synthase-like—supported ammonia assimilation and amino-acid redistribution, consistent with the gill’s frontline roles in ammonia excretion, ion regulation, and acid–base balance [35,37,39]. In the hepatopancreas, DEGs enriched in cell-junction integrity and protease inhibition indicated an early adaptive response to maintain structural and proteolytic homeostasis [45]. At this stage, ACP activity was already significantly reduced, while T-AOC and T-SOD remained statistically unchanged and SR did not differ significantly from the control. Thus, at 4 mg/L, growth was sacrificed first to preserve survival, with nitrogen/amino-acid redistribution and structural maintenance forming the primary metabolic cost.

At 8 mg/L, the maintenance burden expanded across both tissues. In gills, enrichment shifted to oxidative phosphorylation, electron transport, NADH/NAD(P)H dehydrogenase activity, and mitochondrial inner-membrane components, with qRT-PCR-validated downregulation of COX3 and NADH dehydrogenase subunit 1, consistent with increased ATP demand and redox pressure under high environmental stress [43,44]. In the hepatopancreas, SAM synthase-like emerged as a predicted hub linked to methyl-donor supply and methylation-related metabolism [48], together with ribosome biogenesis and post-transcriptional regulatory nodes (U3 small nucleolar RNA-associated protein 11, GEM-associated protein 5-like, 39S ribosomal protein L3). Immune and detoxification effectors, including lysozyme-like, trypsin-1-like, chymotrypsin BII, perlucin-like, cytochrome P450, and GST, were strongly induced, alongside cofactor/vitamin and lipid-remodeling pathways. These transcriptional responses coincided with significant reductions in ACP, T-AOC, and T-SOD and with the lowest WGR and SR. Collectively, these key DEGs define a dose-responsive metabolic network in which nitrogen/amino-acid redistribution, energy and redox remodeling, immune/detoxification/cofactor demand, suppressed physiological defense, and reduced growth and survival are functionally connected. This network elevates maintenance costs and diverts amino acids, methyl donors, ATP, and reducing equivalents away from biomass production [5,26,46,50]. It also highlights candidate biomarkers such as GS, COX III, NADH dehydrogenase subunit 1, SAM synthase, lysozyme, and CYP450/GST for health monitoring under chronic nitrite stress [46,47,48,49,50]. However, the cofactor/vitamin and tryptophan–kynurenine inferences are based on transcriptional enrichment only, and the PPI hubs are predicted candidates rather than confirmed functional drivers. Metabolomic, proteomic, and enzymatic validation is therefore required to determine whether these transcriptional changes translate into functional metabolic reprogramming and to refine nitrite threshold management in *P. monodon* aquaculture.

## 5. Conclusions

This study used *P. monodon* as a model to systematically characterize growth phenotypes, physiological functions, and metabolic network responses under a 30-day chronic exposure regime. The results showed a marked decline in production performance in the exposed groups, with reduced final body weight and weight gain rate accompanied by a downward trend in survival, indicating a substantial “growth cost” even under sustained sublethal stress. Physiologically, hepatopancreas acid phosphatase activity was significantly suppressed, and at the higher exposure level both total antioxidant capacity and total superoxide dismutase decreased significantly, suggesting impairment of immune-related enzymatic functions and antioxidant defenses. Transcriptomic profiling revealed pronounced tissue specialization: gills primarily adjusted nitrogen assimilation and amino acid metabolic allocation under moderate stress, but shifted toward remodeling of the mitochondrial respiratory chain, oxidative phosphorylation, and transmembrane transport under higher stress. In contrast, the hepatopancreas exhibited stronger transcriptional reprogramming, highlighting regulation of cell junction integrity and proteolytic balance, and at high concentrations further intensifying pathways related to redox homeostasis, cofactor and vitamin provisioning, lipid metabolism, and detoxification/immune processes. Key candidate genes and network modules further pinpointed a coordinated axis of “nitrogen metabolism–energy metabolism–vitamin/cofactor supply” as a central driver of elevated maintenance costs during chronic stress. Collectively, these findings provide mechanistic evidence to support chronic nitrite nitrogen risk assessment and threshold management in aquaculture, and offer testable molecular targets for biomarker-based health monitoring and intervention strategies.

## Figures and Tables

**Table 1:** Comparison of growth performance indices of *P. monodon* among experimental groups.

Group	Survival Rate (SR, %)	Initial Mean Body Weight (W0, g)	Final Mean Body Weight (Wt, g)	Weight Gain Rate (WGR, %)
Control	58.89 ± 9.58 ^a^	2.17 ± 0.05 ^a^	3.97 ± 0.24 ^a^	82.98 ± 11.40 ^a^
4 mg/L	51.11 ± 9.82 ^ab^	2.28 ± 0.09 ^a^	3.17 ± 0.17 ^ab^	39.33 ± 9.36 ^b^
8 mg/L	38.89 ± 7.53 ^b^	2.20 ± 0.02 ^a^	2.95 ± 0.16 ^b^	33.86 ± 6.16 ^b^

Values are presented as mean ± SD. Different superscript letters within a column indicate significant differences among groups (*p* < 0.05).

**Table 2:** Effects of nitrite nitrogen on phosphatase activities in *P. monodon*.

Group	Acid Phosphatase (ACP, U/mgprot)	Alkaline Phosphatase (AKP, U/mgprot)
Control	0.25 ± 0.03 ^a^	0.57 ± 0.10 ^a^
4 mg/L	0.13 ± 0.02 ^b^	0.35 ± 0.13 ^a^
8 mg/L	0.16 ± 0.02 ^b^	0.37 ± 0.15 ^a^

Values are presented as mean ± SD. Different superscript letters within a column indicate significant differences among groups (*p* < 0.05).

**Table 3:** Effects of nitrite nitrogen on antioxidant indices in *P. monodon*.

Group	Total Antioxidant Capacity (T-AOC, U/mgprot)	Total Superoxide Dismutase (T-SOD, U/mgprot)
Control	1.65 ± 0.70 ^a^	142.14 ± 31.48 ^a^
4 mg/L	1.17 ± 0.99 ^ab^	104.62 ± 25.60 ^ab^
8 mg/L	0.83 ± 0.16 ^b^	46.10 ± 34.81 ^b^

Values are presented as mean ± SD. Different superscript letters within a column indicate significant differences among groups (*p* < 0.05).

## Data Availability

The raw mRNA sequencing reads have been submitted to the Genome Sequence Archive (GSA) at the National Genomics Data Center, China National Center for Bioinformation, and are accessible under accession number CRA038789.
